# Comparing Prescribing and Dispensing Data of the PCORnet Common Data Model Within PCORnet Antibiotics and Childhood Growth Study

**DOI:** 10.5334/egems.274

**Published:** 2019-04-12

**Authors:** Pi-I D. Lin, Matthew F. Daley, Janne Boone-Heinonen, Sheryl L. Rifas-Shiman, L. Charles Bailey, Christopher B. Forrest, Casie E. Horgan, Jessica L. Sturtevant, Sengwee Toh, Jessica G. Young, Jason P. Block, on behalf of the PCORnet Antibiotics and Childhood Growth Study Group

**Affiliations:** 1 Department of Population Medicine, Harvard Medical School and Harvard Pilgrim Health Care Institute, Boston, MA, US; 2 Institute for Health Research, Kaiser Permanente Colorado, Aurora, CO, US; 3 Oregon Health and Science University, School of Public Health, Portland, OR, US; 4 Applied Clinical Research Center, Children’s Hospital of Philadelphia, Philadelphia, PA, US; 5 For key investigators and stakeholders in the PCORnet Antibiotics and Childhood Growth Study Group, see the Appendix

**Keywords:** Electronic health records, drug prescription, pharmacy dispensing, Common Data Model, distributed research network, oral antibiotic, pediatric

## Abstract

Researchers often use prescribing data from electronic health records (EHR) or dispensing data from medication or medical claims to determine medication utilization. However, neither source has complete information on medication use. We compared antibiotic prescribing and dispensing records for 200,395 patients in the National Patient-Centered Clinical Research Network (PCORnet) Antibiotics and Childhood Growth Study. We stratified analyses by delivery system type [closed integrated (cIDS) and non-cIDS]; 90.5 percent and 39.4 percent of prescribing records had matching dispensing records, and 92.7 percent and 64.0 percent of dispensing records had matching prescribing records at cIDS and non-cIDS, respectively. Most of the dispensings without a matching prescription did not have same-day encounters in the EHR, suggesting they were medications given outside the institution providing data, such as those from urgent care or retail clinics. The sensitivity of prescriptions in the EHR, using dispensings as a gold standard, was 99.1 percent and 89.9 percent for cIDS and non-cIDS, respectively. Only 0.7 percent and 6.1 percent of patients at cIDS and non-cIDS, respectively, were classified as false-negative, i.e. entirely unexposed to antibiotics when they in fact had dispensings. These patients were more likely to have a complex chronic condition or asthma. Overall, prescription records worked well to identify exposure to antibiotics. EHR data, such as the data available in PCORnet, is a unique and vital resource for clinical research. Closing data gaps by understanding why prescriptions may not be captured can improve this type of data, making it more robust for observational research.

## Introduction

Studies on utilization of prescription medications increasingly use large datasets, including records on prescribing from electronic health records (EHRs) and dispensing from pharmacies and insurance claims. These data sources offer large sample sizes in real-world clinical settings [1], but they also have limitations. Studies based on prescribing records omit medications given outside of the health system providing data, and validating drug adherence is difficult, if not impossible, with this source of data alone [2]. Pharmacy dispensings commonly omit prescriptions paid with cash or vouchers, as well as medications dispensed at out-of-network pharmacies. Claims data may also be incomplete if patients change insurance payers [1]. Comparing prescription and dispensing/claims data can provide detailed information on relevant gaps in medication data and predictors of missing prescriptions.

New large-scale networks provide opportunities for advancing these comparisons. The National Patient-Centered Clinical Research Network (PCORnet) is a nationwide distributed research network in the U.S. representing over 130 health systems and patient groups with data on more than 100 million patients [3,4]. Within PCORnet, participating health care systems transform their clinical data collected during routine clinical care into a standardized format using a Common Data Model (CDM) to facilitate combining data across institutions from diverse populations, care settings, and computer and EHR systems [5]. The development of the PCORnet CDM was modeled after the U.S. Food and Drug Administration’s Sentinel CDM (https://www.sentinelinitiative.org), and was informed by several other distributed initiatives such as the Health Care Systems Research Network (HCSRN), the Vaccine Safety Datalink, various AHRQ Distributed Research Network projects, and the Standards & Interoperability Framework Query Health Initiative from the Office of the National Coordinator for Health Information Technology (ONC). The PCORnet CDM includes both prescribing and dispensing tables, allowing for comparison of medication information captured by different sources [1].

The PCORnet Antibiotics and Childhood Growth Study (ABX Study) [6,7] is one of the first observational demonstration projects funded to do research within PCORnet, and the first PCORnet study to examine medication utilization in depth. PCORnet includes a number of large health systems with substantial data on children throughout childhood, making it in an important setting to investigate medication use. The network is diverse, with free-standing children’s hospitals, academic medical centers, community hospitals and ambulatory care systems, and federally-qualified health centers across a wide geographic area. Having access to a subset of systems with both prescribing and dispensing data enhances the network’s capacity for medication-related research, especially considering that children might receive care from several institutions, leading to missing data in prescribing tables. This incomplete prescribing data might be worse for antibiotics, considering that children commonly present to urgent care centers for acute illnesses and receive their antibiotics there.

The ABX study created a large pediatric cohort of 681,739 children from 36 health care institutions to assess the effect of early life antibiotic exposure on weight outcomes. For these 36 institutions, prescribing data was uniformly available; thus, the ABX study used prescribing data as its primary source of information on antibiotic exposure. Dispensing data was available from eight institutions; in these institutions, we compared prescribing records with dispensing records and assessed the potential for missing information from the prescribing data.

**Figure 1:**
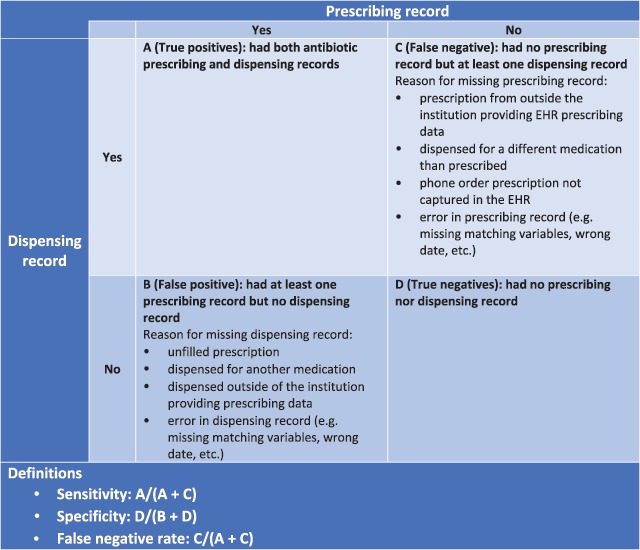
Sensitivity and specificity of using prescribing data to assess antibiotic exposure classification.

In this study, our objectives were to first compare the CDM prescribing and dispensing tables to quantify how well matched they were and to assess characteristics associated with dispensing records that were not captured in the prescribing table. We then examined the extent to which antibiotic exposure classification (exposed vs. non-exposed) is sensitive to the data used. We conducted sensitivity and specificity analyses using dispensing data as the reference and additionally examined characteristics of misclassified patients (Figure 1).

## Methods

### Study population

The ABX study is described in detail elsewhere [6,7]. Briefly, to be included in the cohort, children were required to have at least one same-day height and weight measured in each of three age periods: 1) before 12 months, 2) 12 to 30 months, and 3) after 24 months, ensuring an ongoing connection to the health care institution to more accurately capture antibiotic prescriptions; the overlap between the second and third age periods accommodated children who received their 2-year well-child visit after their second birthday. We limited this study to the eight health care institutions with both prescribing and dispensing information in their CDM tables. Four of the eight institutions were closed integrated delivery systems (cIDS) that provide both health insurance and patient care to patients. The other four institutions are open integrated delivery systems or non-integrated delivery systems where there is no link or only a partial link between care delivery and insurance, referred to as non-cIDS for this study. These systems may capture fewer dispensings as a result.

The study period was from March 2004 to November 2016, with most data captured from 2009 on because this was the start date for the CDM for most PCORnet institutions. We had data available on patients’ prescribing and dispensing records from age 0 to 132 months, although about 90 percent of records were from age 0 to 60 months (Supplementary Material, Table S1). The analysis included 178,785 children from the eight institutions that provided both prescribing and dispensing data. The Institutional Review Boards from each of the contributing institutions and Harvard Pilgrim Health Care approved the study.

### Data Source

Health care institutions standardized their data into the PCORnet CDM for the purpose of interoperability and consistency across systems [5]. Prior to receiving any data, the PCORnet Coordinating Center performed basic data curation and data quality checks to ensure appropriate capture of data elements by institutions and high-level conformance with the CDM specifications [8]. This study utilized CDM version 3.0 which included 15 tables and over 100 variables (http://www.pcornet.org/pcornet-common-data-model/). The ABX study collected data from the prescribing and dispensing tables for oral antibiotics and intramuscular ceftriaxone and penicillin [6], as well as variables from other tables including demographics, procedures, encounters, diagnoses, and vital measures.

### Prescribing and dispensing data

The PCORnet CDM prescribing table captures details regarding provider orders for prescription medications in EHRs. PCORnet uses the National Library of Medicine’s RxNorm classification system for coding prescribing data [9]. RxNorm covers all prescription and many over-the-counter medications available in the United States and provides coding at the ingredient, strength, drug form, and brand name levels. We extracted patient ID (PATID), RxNorm concept unique identifiers (RXNORM_CUI) [9], and medication name (RAW_RX_MED_NAME) from the prescribing table. The PCORnet CDM dispensing table contains outpatient pharmacy dispensing records, such as prescriptions filled through a pharmacy with a claim paid by an insurer. Dispensing data is standardized into FDA National Drug Classification (NDC) terminology which can be mapped to RxNorm, allowing for comparison. From this table, we extracted patient ID (PATID) and National Drug Code (NDC). Table S2 (Supplementary Material) summarizes the source of dispensing from each institution, which included dispensing records from in-house pharmacies, linked insurance claims and/or with national prescribing data sources such as Surescripts [10]. The list of RxNorm concept unique identifiers and NDC codes included in the study is presented in Table S3.

### Data management and cleaning

To characterize each medication order in detail, we assigned antibiotic spectrum (broad vs. narrow), class, and medication name to each prescribing and dispensing record. We defined penicillin, amoxicillin, and dicloxacillin as narrow-spectrum and all others, including penicillin combinations such as amoxicillin/clavulanic acid, as broad-spectrum. We de-duplicated same-day records for each patient, giving priority to the broadest spectrum antibiotic prescribed or dispensed. The final analysis included a total of 15 classes of antibiotics (Supplementary Material, Table S3).

We assigned a same-day encounter type to each prescribing and dispensing record using the encounter type (ENC_TYPE) from the CDM encounter table. Because PCORnet institutions have differential access to prescriptions delivered while inpatient or in the emergency department (ED), we excluded records from these encounters, leaving behind prescriptions that were given in ambulatory settings or unknown/missing settings.

We matched each prescribing record to a dispensing record of the same patient and same medication specification (spectrum, class, medication name) that occurred within seven days after the prescribing record and performed similar procedures to match dispensing records to prescribing records. When one-to-many matching occurred, e.g. multiple prescribing records corresponded to a single dispensing record (<0.4 percent), we kept a single match that had the smallest number of days between prescribing and dispensing records. We assigned a binary variable (1 as match and 0 as no-match) to each prescribing and dispensing record to indicate if the record had a matching record.

We included demographic variables of age, sex (male, female), race (Asian, Black/African American, White, Others, and Unknown), Hispanic ethnicity (yes or no/unknown); and clinical variables of chronic illness [yes or no, defined as 2 or more International Classification of Diseases, 9th Revision, Clinical Modification (ICD-9-CM) codes for a complex condition <72 months of age, based on a previously published code set [6,11]], asthma diagnosis (2 or more ICD-9-CM codes for asthma <72 months), preterm status (any preterm ICD-9-CM code for prematurity <24 months), and evaluated level of health care utilization using total count of encounters of any type (0–132 months). We extracted infection diagnoses that occurred on the same day of prescribing or dispensing records and classified them into Tier 1–3 using the method described by Fleming-Dutra and *et al* [12], where Tier 1 diagnoses were infections that nearly always require antibiotics (e.g., pneumonia), Tier 2 were more antibiotic-discretionary conditions (e.g., suppurative otitis media), and Tier 3 rarely required antibiotics (e.g., nonsuppurative otitis media). Our data capture for disease and clinical conditions occurred before the date of mandatory implementation of ICD-10 (October 2015). Detailed information on the curation of each variable is described elsewhere [6,7].

### Analysis

In the first part of the analysis, we directly compared prescribing and dispensing records. We calculated the percent of records with matching prescribing or dispensing records, counts of prescribing and dispensing records for each patient, and calculated the agreement in the counts of prescribing and dispensing records (0, 1, 2, 3, 4+) per patient using weighted Kappa statistics [13]. We classified the Kappa statistics using the Landis and Koch definitions of poor (κ < 0.20), fair (κ = 0.21–0.40), moderate (κ 0.41–0.60), good (κ = 0.61–0.80), and very good (κ = 0.81–1.00) agreement [14]. We then used mixed-effects logistic regression models to examine characteristics associated with dispensing records that did not have a matching prescribing record, which may suggest the type of antibiotic exposure the prescribing table failed to capture. The logistic regression model calculated the odds of a dispensing record having no matched prescribing record controlling for age, sex, race, Hispanic ethnicity, infection diagnosis tier, and antibiotic spectrum, and accounted for repeated measures per patient and clustering by institution. Multicollinearity between variables was assessed by Variance Inflation Factors, and no strong correlation was found between variables. A significance level of <0.05 was considered statistically significant.

In the second part of the analysis, we examined the impact of exposure misclassification from completely uncaptured antibiotic prescribing records at the patient-level (instead of the prescription record level), which may happen if patients were getting their primary care outside of the PCORnet institutions from which we captured data, or if they received all antibiotics from urgent care or retail clinics. We assessed the sensitivity and specificity of using prescribing data to classify whether a patient ever had an antibiotic prescription using all available data (0–132 months). We defined true positives as patients with at least one prescribing record and one dispensing record; true negatives as patients with no prescribing and no dispensing record; and false-negative as patients with no prescribing record but at least one dispensing record (Figure 1). The sensitivity is the fraction of patients who had at least one antibiotic dispensing record and were correctly identified as exposed to antibiotics by the prescribing table, while the specificity is the fraction of patients without antibiotic dispensing records correctly identified as non-exposed to antibiotics by the prescribing table. For non-cIDS sites, we could not calculate false positive reliably (and therefore specificity), because some of these sites may not have had complete dispensing records due to use of national data sources such as Surescripts; these sources are comprehensive but miss dispensings from pharmacies not linked to Surescripts. We only reported the sensitivity for non-cIDS institutions. We then used multivariable mixed-effect logistic regression with institution as a random effect to examine characteristics of exposed patients who were misclassed as non-exposed (false-negatives). The model included all patients and calculated the odds of being misclassified adjusting for sex, race, Hispanic ethnicity, health status (complex chronic condition, preterm, and asthma), and utilization of health services, measured as total number of encounters.

All analyses were stratified by cIDS and non-cIDS and performed using SAS statistical package version 9.4 (SAS Institute, Cary, North Carolina).

## Results

### Study population and comparison between prescribing and dispensing data

Among the 20,395 pediatric patients included in the study, 48.6 percent were female, 14.2 percent were Black or African-American, and 25.8 percent were Hispanic. The analysis included 537,762 prescribing records and 429,395 dispensing records from ambulatory visits or other non-inpatient and non-ED visits. Most prescribing and dispensing records were for children 24–<60 months of age and were associated with Tier 2 infection diagnoses, and narrow-spectrum antibiotics (Supplementary Material, Table S1). The median number of prescribing records per patient was 2 (Interquartile range, IQR = 1–4) at cIDS and 1 (IQR = 0–3) at non-cIDS; the median number of dispensing records per patient was 2 (IQR = 1–4) at cIDS and 0 (IQR = 0–2) at non-cIDS. Weight kappa coefficient for the counts of prescribing and dispensing records per patient showed very good agreement for patients from cIDS (κ = 0.90) and fair agreement for patients from non-cIDS (κ = 0.39) (Table 1). Comparable results were found by age group (data not presented).

**Table 1:** Description of prescribing and dispensing records.

	cIDS	Non-cIDS

*Total patients, N*	84,566	115,829
*Prescribing records*		
Total prescribing records, N	271,312	266,450
Prescribing records per patient, Median (IQR)	2 (1–4)	1 (0–3)
Prescribing records with matching dispensing records, N (%)^†^	245,471(90.5%)	104,886(39.4%)
*Dispensing records*		
Total dispensing records, N	264,685	164,710
Dispensing records per patient, Median (IQR)	2 (1–4)	0(0-2)
Dispensing records with matching prescribing records, N (%)^†^	245,349(92.7%)	105,348(64.0%)
*Agreement between counts of prescribing and dispensing records, weighted kappa* κ (*95% CI*)^‡^	0.91(0.91–0.91)	0.39(0.38–0.39)

^†^ Matched on patient ID, medication specifications (antibiotic spectrum, class and specific name) and 7 days between records, did not restrict on encounter type. ^‡^ Interpretation for Kappa statistics: poor (κ < 0.20), fair (κ = 0.21–0.40), moderate (κ 0.41–0.60), good (κ = 0.61–0.80), and very good (κ = 0.81–1.00) agreement.

At cIDS, 90.5 percent of prescribing records had matching dispensing records and 92.7 percent of dispensing records had matching prescribing records (Table 1). There were 19,336 (7.3 percent) dispensing records with no matching prescribing records; missing prescription records were more likely for children with African American race (vs. White, OR 1.43, 95 percent CI 1.36–1.49), female (vs. male, OR 1.09, 95 percent CI 1.06–1.12), missing same-day encounters (vs. ambulatory visits, OR 5.28, 95 percent CI 5.03–5.54), Tier 3 diagnoses (vs. Tier 1, OR 1.15, 95 percent CI 1.08–1.23), and if the antibiotic was classified as narrow spectrum (vs. broad spectrum, OR 1.39, 95 percent CI 1.35–1.44) (Table 2). Fewer missing prescription records were evident from 60 to ≤132 months (vs. 24 to <60 months, OR 0.86, 95 percent CI 0.82–0.91) and for Tier 2 diagnoses (vs. Tier 1, OR 0.43, 95 percent CI 0.41–0.46).

**Table 2:** Multivariable logistic regression of factors associated with dispensing records with no matching prescribing records.

	cIDS	Non-cIDS

Total dispensing records, N	264,685	122,770
Dispensing records with no matching prescribing record, N	19,336	17,422
**Adjusted OR of dispensing records having no matching prescribing record (95% CI)^†^**		

Race (vs. White)		
Asian	0.95(0.89, 1.00)	**0.89(0.84, 0.95)**
Black or African American	**1.43(1.36, 1.49)**	**1.34(1.29, 1.38)**
Other	0.94(0.87, 1.02)	**1.92(1.80, 2.05)**
Unknown	1.03(0.97, 1.09)	1.02(0.97, 1.08)
Hispanic (vs. non-Hispanic)	1.02(0.96, 1.08)	**0.63(0.61, 0.66)**
Female (vs. male)	**1.09(1.06, 1.12)**	**1.07(1.04, 1.09)**
Age category (vs. 24 to <60 months)		
0 to <6 months	**1.31(1.23, 1.39)**	1.03(0.98, 1.09)
6 to <12 months	1.02(0.97, 1.07)	0.99(0.96, 1.03)
12 to <24 months	**1.09(1.05, 1.13)**	0.98(0.95, 1.01)
60 to ≤132 months	**0.86(0.82, 0.91)**	**0.88(0.84, 0.92)**
Encounter Type (vs. ambulatory visits)		
Missing same-day encounter	**5.28(5.03, 5.54)**	**10.95(10.53, 11.39)**
Diagnosis (vs. Tier 1)		
Tier 2	**0.43(0.41, 0.46)**	**0.81(0.76, 0.85)**
Tier 3	**1.15(1.08, 1.23)**	0.99(0.93, 1.06)
Missing/Others/Unknown	0.97(0.91, 1.03)	**1.10(1.04, 1.16)**
Narrow Spectrum (vs. Broad Spectrum)	**1.39(1.35, 1.44)**	**0.95(0.93, 0.97)**

^†^ Multivariable logistic regression comparing dispensing records with and without matching prescribing records, adjusting for sex, race, Hispanic ethnicity, age, diagnosis, and antibiotic spectrum and corrected for clustering by institution. Values in bold indicate statistical significance, P < 0.05.

At non-cIDS, 39.4 percent of prescribing records had matching dispensing records and 64.0 percent of dispensing records had matching prescribing records (Table 1). Missing prescribing records (36 percent) were more likely for children of African American race (vs. White, OR 1.34, 95 percent CI 1.29–1.38) and other race (vs. White, OR 1.92, 95 percent CI 1.80–2.05), female (vs. male, OR 1.07, 95 percent CI 1.04–1.09), missing same-day encounter (vs. ambulatory visits, OR 10.95, 95 percent CI 10.53–11.39), missing/other/unknown infection diagnoses (vs. Tier 1, OR = 1.10, 95 percent CI 1.04–1.16); these were less likely for children with Asian race (vs. White, OR 0.89, 95 percent CI 0.84–0.95), Hispanic ethnicity (vs. non-Hispanic, OR 0.63, 95 percent CI 0.61–0.66), Tier 2 diagnoses (vs. Tier 1, OR 0.81, 95 percent CI 0.76–0.85), and narrow-spectrum antibiotics (vs. broad spectrum, OR 0.95, 95 percent CI 0.93–0.97) (Table 2).

### Misclassification of antibiotic exposure at the patient level

Out of 84,566 and 115,829 patients from cIDS and non-cIDS, only 0.7 percent and 6.1 percent, respectively, were misclassified as receiving no antibiotic prescription but had at least one dispensing record, i.e. false negatives. The sensitivity of using prescribing data to classify antibiotic exposure was 99.1 percent and 89.9 percent for cIDS and non-cIDS, respectively. The specificity was 85.9 percent for cIDS (Table 3). At cIDS, males (vs. female, OR 1.43, 95 percent CI 1.19–1.72), patients with a complex chronic condition (OR 1.55, 95 percent CI 1.12–2.22) and patients with asthma (OR 3.44, 95 percent CI 2.72–4.36) had higher odds of being misclassified as non-exposed, whereas those with a higher number of total encounters had lower odds of being misclassified (Q4 vs. Q1, OR 0.24, 95 percent CI 0.18–0.32). At non-cIDS, patients from Asian, African American and other races, with a complex chronic condition, were preterm, and had asthma, indicated higher odds of being misclassified as having no exposure, while patients with unknown race, Hispanic ethnicity, and had the highest level of health care utilization had lower odds of being false-negatives. (Table 3).

**Table 3:** Classification of antibiotic exposure (yes/no) using the prescribing records compared to dispensing records as reference.

Description	cIDS	Non-cIDS

*Total patients, N*	84,566	115,829
*Antibiotic Exposure Classification, N* (*% Total*)^†^		
True-positives	64,187 (75.9%)	42,696(36.9%)
True-negatives	17,793(21.0%)	34,754(30.0%)
False-negatives	589(0.7%)	7,012(6.1%)
*Sensitivity*	99.1%	85.9%
*Specificity*	89.9%	—
***Adjusted OR of false-negative (95% CI)***^‡^		
Male (vs. Female)	**1.36(1.15, 1.61)**	1.04(0.99, 1.09)
Race (vs. White)		
Asian	1.10(0.83, 1.44)	**1.31(1.16, 1.47)**
Black or African American	1.14(0.89, 1.46)	**2.03(1.89, 2.17)**
Other	0.87(0.59, 1.28)	**2.11(1.89, 2.35)**
Unknown	1.13(0.85, 1.50)	**0.88(0.80, 0.96)**
Hispanic (vs. non-Hispanic)	0.97(0.74, 1.29)	**0.58(0.55, 0.62)**
Complex chronic condition (yes vs. no)	**1.58(1.12, 2.22)**	**1.26(1.09, 1.45)**
Preterm (yes vs. no)	1.24(0.97, 1.58)	**1.44(1.30, 1.58)**
Asthma (yes vs. no)	**3.44(2.72, 4.36)**	**1.15(1.03, 1.27)**
Total number of encounters (vs. Q1, 1–20)		
Q2 (21–30)	0.84(0.66, 1.06)	**1.31(1.23, 1.39)**
Q3 (31–45)	**0.68(0.54, 0.87)**	**1.11(1.03, 1.19)**
Q4 (46+)	**0.24(0.18, 0.32)**	**0.77(0.70, 0.85)**

^†^ Antibiotic exposure classification identified by prescribing data with dispensing data as reference. True positives are patients with at least one prescribing record and one dispensing record; true negatives are patients with no prescribing record and no dispensing record; false-negatives are patients with no prescribing record but at least one dispensing record. ^‡^ Multivariable logistic regression of false-negative adjusting for sex, race, Hispanic ethnicity, health status (complex chronic condition, preterm, and asthma), and degree of health system utilization (total number of encounters) and corrected for clustering by institution. Values in bold indicate statistical significance, P < 0.05.

## Discussion

PCORnet facilitates large distributed network research across diverse populations by working with network partners to integrate, primarily, EHR data into a standardized CDM. Having access to EHR data across many institutions facilitates research on large, diverse populations, with the availability of extensive objective data, such as vitals and labs. The available prescription data is also an important asset, but its use requires confidence that it accurately reflects medication utilization. Dispensing data might provide more comprehensive data on utilization if available, and we compared prescribing and dispensing direct across eight heterogeneous health care institutions. In a pediatric population, we found that 90.5 percent and 39.4 percent of antibiotic prescriptions from cIDS and non-cIDS institutions, respectively, had matching dispensing records, while the matching rates for dispensing records were 92.7 percent and 64.0 percent. We found very good agreement in the counts of prescribing and dispensing records at cIDS (κ = 0.91), but only fair agreement for non-cIDS (κ = 0.39). The less robust agreement in non-cIDS sites likely arose because of less comprehensive capture of dispensing, which relied on a variety of sources (e.g., national data from pharmacies and insurance claims) not under the control of the health care institutions. Further, patients receiving care at non-cIDS sites, due to their more open structure, might receive care at multiple institutions, especially for urgent health care needs, such as infections.

Our analyses assessed which antibiotic orders were not captured by prescribing data rather than vice versa. We did so because prescribing data is the primary source of medication information available for EHR-related studies. Overall the proportion of complete missingness was low with uncaptured prescriptions more common if given to children of minority racial groups, female sex, and with diagnoses such as asthma, preterm and complex chronic conditions. The association with these diagnoses might suggest that children with more complex medical histories might seek care at multiple institutions, thus leading to more sporadic capture of their medications. These sources of bias should be considered carefully in this research on medication utilization. Uncaptured prescriptions were less common when a same-day encounter was evident in the EHR. This was predicted since uncaptured prescriptions are often medications given outside the institution providing data, such as those from urgent care or retail clinics.

Missing information on antibiotic use in prescribing data can also arise for other reasons. Changes made to medications after the initial prescription may not be captured in the prescribing table. For example, providers may call a pharmacy to change an antibiotic prescription after obtaining new information post-visit (e.g., allergies, test results); such a change may not be reflected in the EHR. This cause of missing or incorrect data is likely not of major consequence. We examined whether patients in cIDS institutions filled their antibiotic prescriptions, as written, by comparing against dispensing data, and found that only 2 percent of patients received different antibiotics than initially prescribed. Errors in EHR systems may also result in missing information but is likely not a large contributor either. A retrospective survey of computerized prescription at outpatient clinics of tertiary academic medical centers between 1996–2002 found that errors in computerized prescription was 4.9 percent [15] while another observational study on medication dispensing reported rare errors in dispensing (<1 percent) [16].

Our analysis compared the data quality between cIDS and non-cIDS. Compared to non-cIDS, cIDS institutions usually have more comprehensive information on dispensing because these data are integrated with EHR data in the same electronic system. Because of the tightly structured system, patients from cIDS tend to receive their medical care and pharmacy services in the same health care institution, leading to near complete capture of patents’ medication prescriptions and dispensing. In contrast, each non-cIDS institution has a different method of capturing dispensing data, and quality largely depends on the data sources. For example, some non-cIDS institutions purchased commercial databases, primarily Surescripts, to get all pharmacy dispensing for their insured patients, but the dispensing data was only captured if a patient received their prescription at certain pharmacies and had a subsequent clinical encounter within a certain window of time. This restricted availability of dispensing information may lead to accumulation of data only on patients who have more comorbidities requiring frequent office visits. If the dispensing data was only available for patients with certain insurance coverage, because of existing agreements between health care systems and insurers to provide this data, it may have biased the population toward those who are healthier and more affluent [2,17]. These factors should be accounted for in study designs and analyses to assess the quality of the dispensing data.

Based on results from eight institutions, the PCORnet CDM prescribing data had high sensitivity in identifying children with antibiotic prescriptions. Patients with fewer encounters in the EHR systems, a complex chronic condition and asthma were more commonly misclassified as false-negative, but the extent of exposure misclassification was small. To better characterize the bias introduced by this measurement error, we can examine the correlation structure between the measurement error of exposure and of the outcome (dependent vs. independent measurement error) and assess whether the misclassification occurs differentially or non-differentially across outcome groups. Bross demonstrated that for binary exposures and outcomes, independent nondifferential exposure measurement error could only bias the effect estimate of a test toward the null [18]. Simulation studies have also shown that absolute nondifferential misclassification of a binary exposure (use vs. non-use) would, on average, generate bias in effect estimates toward the null [19], though the direction of bias could be in either direction if the exposure is polytomous, or has more than two categories [20]. In the main ABX study, there was no evidence to suggest that the binary outcome, which was overweight at age 5 (BMI z-score ≥ 85^th^ percentile, yes vs. no), was differentially measured among children misclassified for their binary antibiotic exposure status [7]. Thus, we expect the exposure misclassification in this study biased estimates toward the null. For exposures with more information, such as the count of prescriptions, or with more outcome categories, determining the direction of the bias is more difficult. In this case, a comprehensive sensitivity analysis using the dispensing data as the source of the exposure may be necessary to fully understand the magnitude and direction of bias from missing prescribing information.

Because of their participation in a large research network, PCORnet institutions may have better curated and more complete prescription records than institutions that are not part of large networks and have not had the opportunity to undergo repeated data quality assessments. Results of our study generally demonstrated the robustness of the PCORnet CDM prescribing data for research, using data from diverse health care systems and varied sources of dispensing information. Because antibiotics have a high prescription filling rate (93 percent from pediatric primary care [21] and 93 percent from emergency departments [22]), this medication class was a good use case for exploring data quality; medications used for chronic diseases, with lower fill rates, may provide different results. Our data demonstrated that nearly all antibiotics prescribed were dispensed (separate analysis using cIDS, data not presented), validating the general assumption of a low unfilled rate for antibiotic prescriptions. Characterizing missing information in the prescribing table by comparison with another data source, such as the dispensing table or self-reported medication use, can be useful in developing an algorithm to better define medication use and assess potential bias, such as using methods of bias analysis [23], multiple imputation [24,25], propensity score calibration [26], or even machine learning [27]. Health Plan Research Networks (HPRN) within PCORnet are also working to perform more data linkage of claims and EHR data providing an opportunity in the future to comprehensively compare data for multiple classes of medication.

Our study did have several limitations. Due to data-sharing limitations, we could not obtain the dates of prescribing and dispensing records; thus, trends of antibiotics prescribing practice and filling over time could not be evaluated. Other studies have noted a decline in the use of antibiotics, especially among the pediatric population [28,29]. We also recognize the potential for unmeasured confounding in the assessment of factors associated with missing records; for example, we did not have data on providers/prescribers, insurance coverage, socioeconomic status, and drug cost [22,30,31,32]. We could not assess patients’ actual medication adherence, i.e., if patients took the medication and did so for the entire period prescribed. Finally, our analyses only focused on antibiotics prescribed to children at ambulatory visits or associated with other/missing encounter types, and the results may not be generalizable to other medications, age groups, or to antibiotics given at inpatient and ED visits.

## Conclusions

Prescribing and dispensing data were well matched in eight health care institutions included in this study. A higher percent of prescribing and dispensing records from cIDS had matching dispensing and prescribing records compared to non-cIDS. Most uncaptured prescriptions did not have same-day encounters in the EHR, suggesting they were medications given outside the institution providing data, such as those from urgent care or retail clinics. The PCORnet CDM prescribing table had good sensitivity in identifying pediatric patients who were exposed to antibiotics. Patients misclassified as false-negatives were more likely to have complex chronic conditions and asthma. The findings from this study may be used to facilitate future development of an algorithm to more accurately define antibiotic use among the pediatric population, by integrating individual-level and system-level predictors of missing medication use. EHR data, such as the data available in PCORnet, is a unique and vital resource for clinical research. Closing data gaps by expanding the capture of dispensing records will improve this resource.

## Additional File

The additional file for this article can be found as follows:

10.5334/egems.274.s1 Appendix 1. Members of the PCORnet Antibiotics and Childhood Growth Study Group.
