# Supplementary material for: Comparing Prescribing and Dispensing Data of the PCORnet Common Data Model Within PCORnet Antibiotics and Childhood Growth Study
Source: EGEMS (Wash DC). 2019 Apr 12;7(1):11. doi: 10.5334/egems.274 (PMC6460498; doi:10.5334/egems.274)
Supplement: Appendix 1. — Members of the PCORnet Antibiotics and Childhood Growth Study Group. [file egems-7-1-274-s1.pdf]

Supplementary Material

Appendix 1: Members of the PCORnet Antibiotics and Childhood Growth Study Group

- David Arterburn, MD, Kaiser Permanente Washington Health Research Institute, Seattle, Washington
- Arthur Davidson, MD, MPH, Denver Public Health, Denver, Colorado
- Stephanie L. Fitzpatrick, PhD, Kaiser Permanente Northwest, Portland, Oregon
- Michael Horberg, MD, MAS, Kaiser Permanente Mid-Atlantic Permanente Research Institute, Rockville, Maryland
- Elyse O. Kharbanda, MD, MPH, HealthPartners Institute, Minneapolis, Minnesota
- Pedro Rivera, MS, OCHIN Inc., Portland, Oregon
- Alexander J. Stoddard, MS, Medical College of Wisconsin, Milwaukee, Wisconsin

Table S1. Characteristics of antibiotic prescribing and dispensing records from ambulatory visits (N=20,395 patients)

	**Number of Patients, N (%)**	**Number of Prescribing Records, N (%)**	**Number of Dispensing Records, N (%)**
**Total**	**200,395**	**537,762**	**429,395**
Person-level characteristics			
Race			
White	124,895 (62.3)	272,642 (63.5)	272,642 (63.5)
Asian	13,772 (6.9)	27,117 (6.3)	27,117 (6.3)
Black or African American	29,335 (14.6)	66,311 (15.4)	66,311 (15.4)
Other	8,589 (4.3)	18,772 (4.4)	18,772 (4.4)
Unknown	23,804 (11.8)	44,553 (10.4)	44,553 (10.4)
Hispanic			
No	148,603 (74.2)	343,585 (80.0)	343,585 (80.0)
Yes	51,792 (25.8)	85,810 (20.0)	85,810 (20.0)
Sex			
Male	102,973 (51.4)	284,721 (53.0)	228,299 (53.2)
Female	97,422 (48.6)	253,041 (47.1)	201,096 (46.8)
Institution			
cIDS 1	16,491 (8.2)	43,736 (8.1)	42,650 (9.9)
cIDS 2	24,950 (12.5)	76,061 (14.1)	71,771 (16.7)
cIDS 3	19,660 (9.8)	74,604 (13.9)	74,693 (17.4)
cIDS 4	23,465 (11.7)	76,911 (14.3)	75,571 (17.6)
Non-cIDS 1	9,712 (4.9)	27,782 (5.2)	20,194 (4.7)
Non-cIDS 2	22,923 (11.4)	17,091 (3.2)	20,354 (4.7)
Non-cIDS 3	44,137 (22.0)	157,844 (29.4)	72,090 (16.8)
Non-cIDS 4	39,057 (19.5)	63,733 (11.9)	52,072 (12.1)
Record-level characteristics			
Age category			
0 - <6 months		38,324 (7.1)	28,806 (6.7)
6 - <12 month		99,818 (18.6)	75,341 (17.6)
12 - <24 months		155,264 (28.9)	120,540 (28.1)
24 - <60 months		192,206 (35.7)	159,091 (37.1)
60 - 132 months		52,150 (9.7)	45,617 (10.6)
Encounter type			
Ambulatory visits		419,084 (77.9)	361,296(84.1)
Missing same-day encounter		118,678 (22.1)	68,099(15.9)
Infection Diagnosis			
Tier 1		34,738 (6.5)	30,623 (7.1)
Tier 2		264,572 (49.2)	227,463 (53.0)

Tier 3		46,468 (8.6)	38,220 (8.9)
Other		191,984 (35.7)	133,089 (31.0)
Antibiotic Spectrum			
Broad		248,961 (46.3)	197,844(46.1)
Narrow		288,801 (53.7)	231,551(53.9)

Table S2. Source of dispensing data by institutions

**Institution**	**Source of dispensing**[†]	**Time of recording for dispensing data**	**Capture records of medication dispensing from other sources (e.g. Surescript)?**
cIDS1	Pharmacy dispensing	When the patient picks up the prescription.	Yes
cIDS2	Pharmacy dispensing	When the patient picks up the prescription.	Yes
cIDS3	Pharmacy dispensing	When the patient picks up the prescription.	Yes
cIDS4	Pharmacy dispensing	When the patient picks up the prescription.	Yes
Non-cIDS1	Pharmacy dispensing	When the patient picks up the prescription.	Yes
Non-cIDS2	Pharmacy dispensing	When the patient picks up the prescription.	No
Non-cIDS3	Pharmacy claim	When the pharmacist fills the prescription, and puts on the shelf. Still recorded if the patient never picks up the prescription	Yes
Non-cIDS4	Pharmacy claim	When the patient picks up the prescription.	Yes

[†]Pharmacy dispensing: Any medication dispensed at institutions' pharmacy regardless of the source of payment and the source of prescription (electronic prescription from your health institution, verbally ordered by prescriber, paper prescription, transferred prescription)

Pharmacy Claim: records of medication dispensed using the patients' pharmacy benefit, does not capture out-of-pocket payment

Table S3. List of oral antibiotics included in the study

Class	Medication Names	RxNorm CUI	NDC
1ST GEN CEPHALOSPORIN	CEFADROXIL, CEPHALEXIN	197452, 309042, 309044, 309080, 309081, 309096, 309097, 309098, 313888, 313926, 581583, 581584	00093314501, 00093314505, 00093314701, 00093314705, 00093417573, 00093417574, 00093417773, 00093417774, 00143989701, 00143989705, 00143989801, 00143989805, 16714038901, 42043014001, 42043014105, 42043014238, 42043014258, 42043014338, 42043014358, 63304065601, 63304095801, 63304095802, 63304095901, 63304095902, 65862001801, 65862001805, 65862001901, 65862001905, 65862008301, 67877021901, 67877021905, 67877022001, 68180012101, 68180012102, 68180012201, 68180012202, 68180012301, 68180012302, 68180012401, 68180012402, 68180018102, 68180018203, 76439010110, 76439010150, 76439010250
2ND GEN CEPHALOSPORIN	CEFACLOR, CEFPROZIL, CEFUROXIME	197452, 309042, 309044, 309080, 309081, 309096, 309097, 309098, 313888, 313926, 581583, 581584	00093107573, 00093107673, 00093107678, 00173074000, 00173074100, 00173074110, 00781620246, 00781620257, 00781620346, 00781620357, 00781620391, 16571007012, 16571007112, 16714021502, 16714021503, 16714021601, 16714021602, 16714021603, 16714038603, 16714038701, 16714038702, 16714038703, 16714039603, 16714039701, 16714039702, 16714039703, 42043026338, 42043026357, 42043026377, 57237003501, 63304095602, 63304096304, 63304096403, 63304096404, 65862009901, 65862010001, 65862010075, 65862069960, 67877021660, 68180030220, 68180030320, 68180040101, 68180040102, 68180040103, 68180040201, 68180040202, 68180040203, 68180040301, 68820001815, 68820001816, 68820001817, 68820001915, 68820001916, 68820001917
3RD GEN CEPHALOSPORIN	CEFDINIR, CEFIXIME, CEFPODOXIME, CEFTIBUTEN, CEFTRIAXONE	1043022, 1043025, 1043030, 1373014, 197451, 200346, 2193, 309054, 309058, 309077, 309078, 309079, 309085, 409823, 419849, 476576, 581574, 705008	00074377160, 00093413664, 00093413673, 00093413764, 00093413773, 00781217660, 00781543920, 00781607746, 00781607761, 00781607846, 00781607861, 00781616846, 00781616852, 00781616946, 00781616952, 16714020601, 16714020602, 16714020701, 16714020702, 16714039102, 16714039201, 16714039202, 16714039301, 16714039302, 16714040301, 16714040302, 27437020108, 27437020311, 27437020602, 27437020603, 27437020811, 42043025167, 42043025238, 42043025267, 57237009960, 63304096503, 63304096504, 63304096603, 63304096604, 65862009620, 65862014001, 65862014050, 65862014101, 65862014150, 65862017760, 65862021801, 65862021860, 65862021901, 65862021960, 65862075150, 65862075250, 65862075275, 67253000841, 67253000846, 67253000941, 67253001106, 67253001241, 67253001242, 67253001341, 67253001342, 68001015006, 68180020201, 68180020203, 68180040501, 68180040703, 68180040704, 68180071160, 68180072210, 68180072220, 68180072310, 68180072320, 68820006417, 68820006437, 68820006517, 68820006537
FLUOROQUINOLONE	CIPROFLOXACIN, LEVOFLOXACIN, MOXIFLOXACIN	197511, 197512, 199370, 199884, 199885, 211816, 309308, 309309, 309310, 311296, 311787, 477391, 544445	00045151501, 00045152050, 00085173301, 00085177301, 00085177701, 00143992701, 00172531160, 00172531260, 00172531360, 10147094106, 13668008250, 16252051401, 16252051501, 16252051605, 16571041110, 33342002108, 50383028604, 50383028608, 50383028616, 50419077301, 50419077701, 50458017001, 50458092550, 55111012506, 55111012601, 55111012701, 55111027950, 61442022201, 68180024001, 68180024108, 68180039201, 68180039301, 68382001701
GLYCOPEPTIDE	VANCOMYCIN	239210, 313570, 313571	17478074102, 17478074202, 66593312502

LINCOSAMIDE	CLINDAMYCIN	197518, 284215, 309329, 562266, 748743	00009033102, 00009076004, 00093317101, 00527138104, 00527138201, 00574012901, 00591293201, 00591312001, 00591570801, 00781211201, 59762001601, 59762332801, 59762501002, 63304069201, 63304069205, 63304069301, 64980051110, 65162046819, 65862018501, 65862059601
MACRO/SULFA	ERYTHROSULF	895384	00074715613, 00074803053, 00555044521, 00555044523, 00904247504, 00904247507, 00904247508, 51285044521, 51285044522, 51285044523
MACROLIDE	AZITHROMYCIN, CLARITHROMYCIN, ERYTHROMYCIN	105260, 141963, 197516, 197517, 204844, 205860, 211307, 212446, 240741, 248656, 308459, 308460, 309322, 598006, 686383, 686400, 686402, 686405, 686418, 686420, 749783, 863603	00054003721, 00069306030, 00069311019, 00069312019, 00069313019, 00069314019, 00074318813, 00074374716, 00074374816, 00074630213, 00074630513, 00074630616, 00074636902, 00074636910, 00074637316, 00093202623, 00093202631, 00093202694, 00093202723, 00093714609, 00093714618, 00093714656, 00093714756, 00093714823, 00093714923, 00093714931, 00093714994, 00093715706, 00093715806, 00093716933, 00093716956, 00185720370, 00185721268, 00781149631, 00781149668, 00781602246, 00781602252, 00781602346, 00781602352, 24338010213, 24338012213, 24338013013, 24338013213, 24338013402, 24338013610, 50111076728, 50111078751, 50111078766, 50111079120, 50111079222, 50111079320, 59762306001, 59762306002, 59762306003, 59762311001, 59762312001, 59762313001, 59762314001, 60505258100, 60505258103, 63304082104, 63304082204, 64679096101, 64679096105, 65862022560, 68382076405, 68382076406, 68382076505, 68382076506, 68387056506, 68774030229, 68774030235, 68774030329, 68774030335
NITROFURANS	NITROFURANTOIN	1648755, 207790, 207791, 311989, 311994, 311995, 539712, 7454	00093213001, 00093213010, 00149000705, 00185012201, 00378165001, 00378165005, 00591368401, 43386045011, 47781030301, 47781030601, 47781030701, 47781030801, 52427028501, 52427028601, 57664023932, 59630045008, 59630045016, 65162068988, 66993047173
OTHER	METRONIDAZOLE, TRIMETHOPRIM	199055, 311681, 314106, 562707, 6922	00093085105, 00591252101, 00591252125, 00591252205, 00591252250, 00591396901, 00591396905, 00591396925, 00591397005, 00591521505, 38779014608, 38779023801, 38779023804, 38779023805, 38779023809, 49452472703, 50111033301, 50111033302, 50111033306, 50111033401, 50111033402, 51552003805, 51552046502, 51552046504, 51552046505, 51552046506, 51927155900, 53489013601
OXAZOLIDINONES	LINEZOLID	311345	00009513601, 00054031950
PENICILLIN	AMOXICILLIN, AMPICILLIN, DICLOXACILLIN, PENICILLIN	197595, 197596, 239191, 308177, 308181, 308182, 308188, 308189, 308191, 308192, 308194, 308210, 313797, 313799, 313800, 313850, 313945, 598025, 834040, 834046, 834061, 834102	00003173745, 00003173830, 00003173840, 00003173845, 00029600822, 00029600922, 00029600923, 00029604954, 00029604955, 00029604959, 00093117201, 00093117210, 00093117401, 00093117410, 00093226301, 00093226401, 00093226701, 00093226801, 00093226805, 00093310701, 00093310705, 00093310905, 00093312301, 00093412573, 00093412574, 00093412773, 00093412774, 00093415073, 00093415079, 00093415080, 00093415573, 00093415579, 00093415580, 00093416073, 00093416076, 00093416078, 00093416173, 00093416176, 00093416178, 00093519874, 00093519974, 00143988601, 00143988650, 00143988675, 00143988701, 00143988750, 00143988775, 00143988801, 00143988815, 00143988880, 00143988901, 00143988915, 00143988980, 00143993801, 00143993805, 00143993901, 00143993905, 00143995101, 00143995120, 00172741821, 00172741823, 00172742021, 00172742022, 00781120501, 00781202001, 00781202005, 00781225801, 00781261301, 00781261305, 00781261331, 00781506101, 00781506120, 00781603946, 00781603955, 00781603958, 00781604146, 00781604155, 00781604158, 00781612146, 00781615646, 00781615652, 00781615657, 00781615746, 00781615752,

			00781615757, 16714023401, 16714023501, 16714029801, 16714029802, 16714029904, 43598020750, 43598020751, 43598020752, 43598020952, 43598020953, 43598021901, 43598022350, 43598022351, 43598022352, 49884007247, 49884007269, 49884007327, 49884007328, 49884007347, 54868412902, 55370088714, 57237002801, 57237002901, 57237003001, 57237003005, 57237003101, 57237003105, 57237003201, 57237003301, 57237003350, 57237003375, 57237004001, 57237004101, 57237004105, 57237004199, 59762102003, 59762102202, 59762102204, 59762102207, 59762102304, 59762102305, 59762102306, 59762105002, 59762153401, 63304051501, 63304065501, 63304076020, 63304076101, 63304076120, 63304096901, 63304096903, 63304096904, 63304097001, 63304097003, 63304097004, 65862001501, 65862001601, 65862001705, 65862007101, 65862007150, 65862007175, 65862017501, 65862017601, 67253014150, 67253014215, 67253014308, 67253014310, 67253014315, 67253014646, 67253014647, 67253014845, 67253014846, 67253014847, 67253014943, 67253014944, 67253015050, 67253018010, 67253018310, 67253018320, 67253020010, 67253020011, 67253020110, 67253020111, 67253020150, 67253020210, 67253020220, 67253020310, 67253020320, 68387043040
PENICILLIN COMBINATION	AMOXCLAV	562251, 562508, 617296, 617302, 617304, 617309, 617316, 617322, 617333, 617339, 617423, 617430, 617993	00029608522, 00029608523, 00029608539, 00029608739, 00029609022, 00029609023, 00029609039, 00029609229, 00029609440, 00093227034, 00093227234, 00093227434, 00093227534, 00093227773, 00093227973, 00093867574, 00093867575, 00093867578, 00143985316, 00143985324, 00143985375, 00143998101, 00143998150, 00143998175, 00143998201, 00143998250, 00143998275, 00172740620, 00172740722, 00172740822, 00781161966, 00781164366, 00781185220, 00781187431, 00781610246, 00781610446, 00781613948, 00781613954, 00781613957, 16714029201, 16714029202, 16714029203, 16714029301, 16714029302, 16714029303, 16714029401, 16714029402, 16714029403, 16714029501, 16714029601, 16714029701, 43598000451, 43598000452, 43598001251, 43598001252, 43598001253, 43598020351, 43598020354, 43598020369, 43598020451, 43598020452, 43598020453, 43598020614, 43598020850, 43598020851, 43598020852, 43598021350, 43598021351, 43598021352, 43598021830, 43598022114, 49884016827, 49884016828, 49884020128, 49884020149, 49884020170, 49884029907, 54569433700, 60432006500, 60432006547, 60432006575, 60432007046, 60432007048, 60432007075, 63304076801, 63304076807, 63304097701, 63304097703, 63304097704, 63304097901, 63304097903, 63304097904, 65862050220, 65862050320, 65862053301, 65862053401, 65862053513, 65862053575, 66685100100, 66685100101, 66685100200, 66685101100, 66685101101, 66685101102, 66685101200, 66685101201, 66685101202
SULFA	SULFAMETH/TRIMETH, SULFISOXAZOLE, TRIMETHOPRIM	198332, 198334, 198335, 198420, 208406, 261353, 313134	00004100328, 00093547616, 00179013806, 00179013820, 00472128516, 00472128533, 00591557101, 00603168458, 00603168558, 00603578021, 00603578128, 13310014501, 13551050105, 50383082316, 50383082416, 53489014501, 53489014505, 53489014601, 53489014605, 53746027101, 53746027105, 53746027201, 53746027205, 54879000716, 61971011501, 61971012005, 65862041901, 65862042005, 65862049647
TETRACYCLINE	DOXYCYCLINE, MINOCYCLINE, TETRACYCLINE	1649988, 1650030, 1650142, 1650143, 197984, 197985, 198252, 283535,	00069097065, 00069097193, 00143314150, 00143314205, 00172240760, 00172298570, 00378602101, 00378602389, 00527133601, 00527133850, 00591555350, 10337081403, 49884009304, 49884072601, 53489011802, 53489011905,

		310026, 702296	53489055001, 63304069401, 65862021150, 68180065208
